# Association of obesity with incident atrial fibrillation in Korea and the United Kingdom

**DOI:** 10.1038/s41598-023-32229-9

**Published:** 2023-03-30

**Authors:** Sung Hwa Choi, Pil-Sung Yang, Daehoon Kim, Jung-Hoon Sung, Eunsun Jang, Hee Tae Yu, Tae-Hoon Kim, Hui-Nam Pak, Moon-Hyoung Lee, Gregory Y. H. Lip, Boyoung Joung

**Affiliations:** 1 Division of Cardiology, Department of Internal Medicine, Severance Cardiovascular Hospital, Yonsei University College of Medicine, 50-1 Yonseiro, Seodaemun-gu, Seoul, 03722 Republic of Korea; 2 Department of Cardiology, CHA Bundang Medical Center, CHA University, Seongnam, Republic of Korea; 3 Liverpool Centre for Cardiovascular Science, University of Liverpool and Liverpool Heart and Chest Hospital, Liverpool, UK

**Keywords:** Risk factors, Epidemiology

## Abstract

Obesity has been linked to atrial fibrillation (AF) burden and severity, and epidemiological studies suggest that AF is more prevalent in whites than Asian. We aimed to investigate whether obesity mediates associations with AF in Europe and Asia using patient-level data comparisons of two cohort studies. Using Korean National Health Insurance Service’s Health Screening (NHIS-HealS) and U.K. Biobank data, we included 401,206 Korean and 477,926 British aged 40–70 years without previous AF who received check-ups. The incidence and risk of AF were evaluated regarding different body mass index (BMI) values. The obese proportion (BMI ≥ 30.0 kg/m^2^, 2.8% vs. 24.3%, *P* < 0.001) was higher in the U.K. than the Korean. In the Korean and U.K. cohort, the age- and sex-adjusted incidence rates of AF were 4.97 and 6.54 per 1000 person-years among obese individuals. Compared to Koreans, the risk of AF was higher in the British population, with adjusted hazard ratios of 1.41 (Korea, 95% CI 1.26–1.58) and 1.68 (UK, 95% CI 1.54–1.82) in obese participants (*P* for interaction < 0.05). Obesity was associated with AF in both populations. British subjects had a greater incidence of AF related to the high proportion of obese individuals, especially participants in the obesity category, the risk of AF also increased.

## Introduction

Atrial fibrillation (AF) raises the risks of mortality and morbidity resulting from stroke and congestive heart failure, impairs patient quality of life^1–4^, and increases health care costs^5^. Epidemiological studies suggest that AF is more prevalent in whites than Asian or Afro-Caribbean/other races^6–8^. Based on Medicare data from 2007, the prevalence rates of AF per 100 beneficiaries were 9.08 in whites, 4.63 in blacks, and 4.75 in other/unknown races^9^. The AF incidence rate ranged from 3.3 to 9.9% in Europe, was 9.9/1000 person-years in Rotterdam^10^ and 3.3/1000 person-years in the Netherlands^11^, and ranged from 3.3 to 19.2 per 1000 person-years among predominantly American cohort studies^10,12,13^. In contrast, recently reported AF prevalence rates ranged from 1.1 to 1.6% in Asian countries with projected prevalence rates of 5.4% in South Korea and 4.0% in Taiwan in year 2050^1,4^.

Although the precise reasons behind the differences in AF epidemiology between Asians and white Europeans remain unclear, more prevalent cardiovascular risk factors and diseases, including smoking, obesity, hypertension, ischemic heart diseases, and diabetes, in Western countries may play important roles^14^. Although aging is a major risk factor for AF, the population attributable risks for higher body mass index increases over time^15^. Obesity is one of the modifiable risk factors for AF and has been reported to be related to both AF burden and severity^16,17^. Body mass index (BMI) has been used widely to assess general obesity, which is a major risk factor for cardiometabolic disease and overall death. Also, waist circumference (WC) is a sensitive indicator of body fat distribution that is considered to characterize central obesity^18^. Both WC and waist-to-hip circumference ratio are related to a greater risk for AF^17^. Of note, a J-curve relationship was observed between BMI and the risk of new-onset AF in the Asian population, indicating that abdominal obesity is an important risk factor for AF^17^. If the effect of obesity on new-onset AF has a direct relationship with the continuous increase of BMI, the risk of new-onset AF will be similarly seen in different ethnic populations from different parts of the world.

We therefore hypothesized that the difference in the distribution of BMI and WC can partially explain the ethnic differences in AF incidence. We aimed to investigate whether obesity mediates the association with AF in Europe and Asia using patient-level data comparisons of two cohort studies, employing data from the Korea National Health Insurance Service’s Health Screening (NHIS-HealS) and U.K. Biobank cohorts. The relationships between BMI or WC and incident AF were analyzed in both cohorts.

## Methods

### Study patients

The Korean NHIS-HealS cohort was released in 2015, and the profile for this cohort has been described previously^19^. This cohort consisted of 457,509 Koreans aged 40–80 years old as an initial 2002 cohort who were followed through 2013 to collect data related to lifestyle and behaviors using questionnaires and major results of health examinations. The cohort population was a 10% simple random sample of all health screening participants seen in 2002 and 2003.

**Figure 1:**
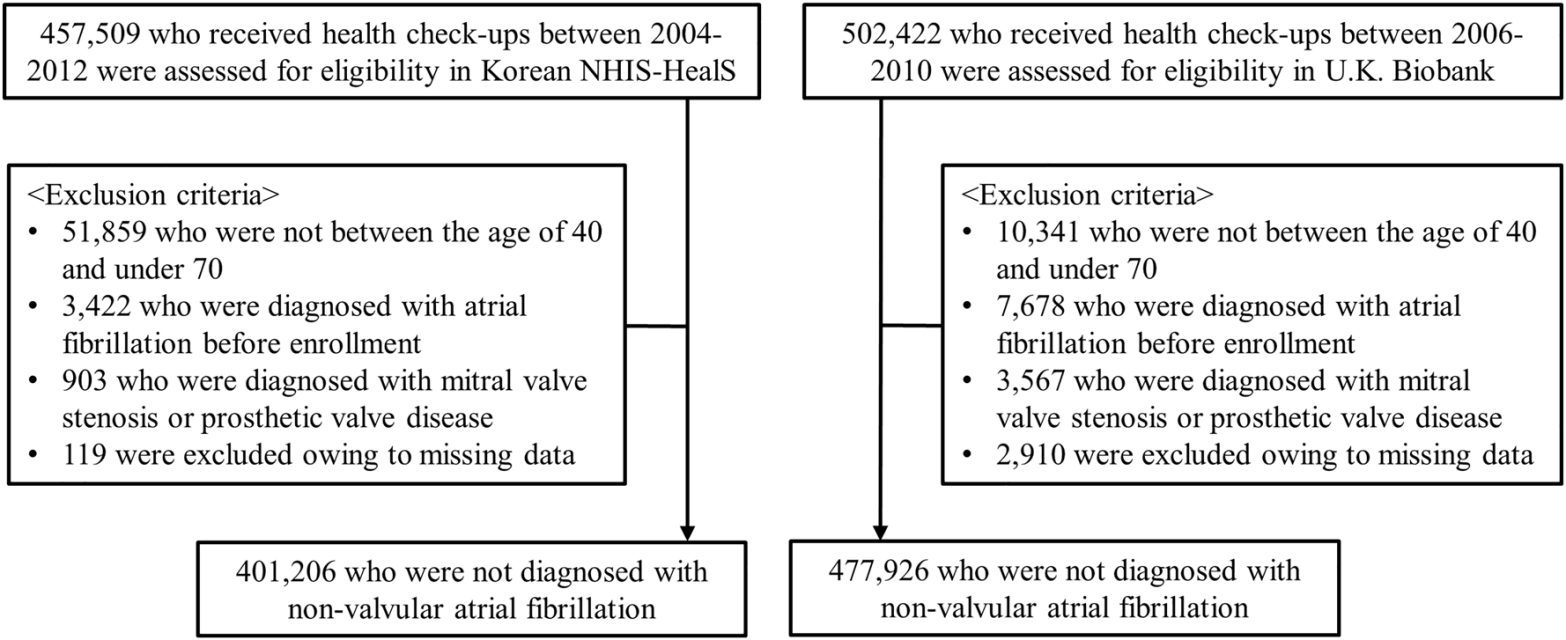
Flow diagram of the study population. Proportions of obese and severely obese individuals were higher in the U.K. Biobank cohort than the Korean NHIS-HealS cohort. *NHIS-HealS* National Health Insurance Service’s Health Screening.

NHIS-HealS is based on information obtained through the national health screening programs of Korea^20^. All insured adults are eligible for a general health screening program that is biennially conducted (annually for manual workers). The participation rate in the general health screening program among the eligible population was 74.8% in 2014. The general health screening program can be applied at least once every 2 years for the entire population of Korean adults aged 40 years or older. The healthcare institutions for screening are designated according to the Framework Act on Health Examinations and must meet the standards of manpower, facilities, and equipment. Two cohort are based on similar enrollment methods. However, because people older than 70 years are included only in the Korean NHIS-HealS cohort, the restriction in age (40–70 years) was used. We excluded patients with the following: (1) those aged < 40 or > 70 years (n = 51,859); (2) those with AF (International Classifications of Diseases, 10th revision [ICD-10] code I48) before the health check-up (n = 3422); (3) those with valvular heart diseases, such as mitral valve stenosis or prosthetic valve status (ICD-10 codes I050, I052, and I342) (n = 903); and (4) those with missing data for BMI or other covariates (n = 119). Overall, 401,206 participants were included (Fig. 1).

The U.K. Biobank recruited 502,422 participants aged 37–73 years from 22 assessment centers across England, Scotland, and Wales from 2007 to 2010. Approximately 9.2 million people aged 40–69 years living within 40 km of one of the 22 assessment centers in England, Wales and Scotland were invited to participate in the cohort, and 5.5% participated in the baseline assessment.

The UK Biobank Study is a large prospective cohort study, established primarily to investigate the genetic and lifestyle determinants of a wide range of diseases of middle and later life^21^. Extensive questionnaire data, physical measurements, and biological samples were collected at recruitment, and there is ongoing enhanced data collection in large subsets of the cohort, including a repeat baseline assessment, genotyping, biochemical assays, Web-based questionnaires, physical activity monitoring, and multimodal imaging. All participants are followed up for health conditions through linkage to national electronic health-related data sets. Details of the study design and data collection have been described previously^22^. The current analysis excluded patients with the following: (1) those who were aged < 40 or > 70 years (n = 10,341); (2) those with AF (ICD-10 code I48) before the health check-ups (n = 7678); (3) those with valvular heart disease (n = 3567); and (4) those with missing data for BMI or other covariates (n = 2910). Overall, 477,926 participants were finally included. To make certain that the new AF diagnosis was actually a new one, we apply look-back period of 3 years in both cohorts.

### Outcome ascertainment

In the Korean NHIS-HealS cohort, new-onset AF was defined as the first occurrence during ≥ 2 different days of hospital visits (outpatient) or the first admission with a diagnosis of AF per the ICD-10 code (I48). The positive predictive value of this was 94.1%^1–3,19,23,24^. In the UK biobank cohort, new-onset AF was established based on more than one hospital-inpatient or two primary care records of ICD-10 codes in the database or self-reported non-cancer illness code (1471, 1483). Detailed definitions of the comorbidities and outcomes are presented in Supplementary Tables S1 and S2.

### Statistical methods

BMI was employed as a continuous and categorical variable in terms of the World Health Organization/National Institutes of Health classification scheme (underweight, BMI < 18.5 kg/m^2^; normal, BMI 18.5–< 25.0 kg/m^2^; overweight, BMI 25.0– < 30.0 kg/m^2^; obese, BMI > 30.0 kg/m^2^; and severely obese, BMI ≥ 40.0 kg/m^2^). WC was also considered as both continuous and categorical variables (abdominal obesity was defined as ≥ 94 [U.K.] or 90 cm [Korea] for men and ≥ 80 cm for women)^17,25^.

Data are expressed with mean ± standard deviation (SD) values for continuous variables and by proportions of categorical variables. Student’s *t*-test was employed for the comparison of continuous variables. The chi-squared test was employed to analyze categorical variables. One-way analysis of variance and the chi-squared test or Fisher’s exact test as a post-hoc test for each BMI group were used to compare continuous variables.

We examined the association between BMI, WC, and the risk of developing new-onset AF using a Cox proportional hazards regression method. Covariates selected for adjustment included age; sex; history of hypertension, diabetes mellitus, dyslipidemia, congestive heart failure, chronic kidney disease (CKD), or end-stage renal disease (ESRD); previous myocardial infarction (MI), transient ischemic attack, or stroke; alcohol use; and former or current smoker. Estimating new-onset AF, we employed the Find and Gray method to regard death as a competing risk^26^. The proportional hazards assumption test based on the scaled Schoenfeld residuals was analyzed for inspection of the proportional assumption for the Cox regression.

Two-sided *P* values < 0.05 were considered statistically significant. Statistical analyses were conducted using R version 4.1.2 (www.R-project.org; The R Foundation for Statistical Computing, Vienna, Austria).

### Ethical approval

This study was approved by the institutional review board (IRB) of Yonsei University Health System (4-2022-0341), and the informed consent requirement was waived. The UK Biobank study has approval from the North West Multi-center Research Ethics Committee (REC approval 21/NM/0157) This research was conducted using the UK Biobank resource under application 77793. Informed consent was obtained by UK Biobank for all participants. Participants who had withdrawn consent following initial enrolment were excluded from the analysis. All research followed the relevant regulations.

## Results

**Figure 2:**
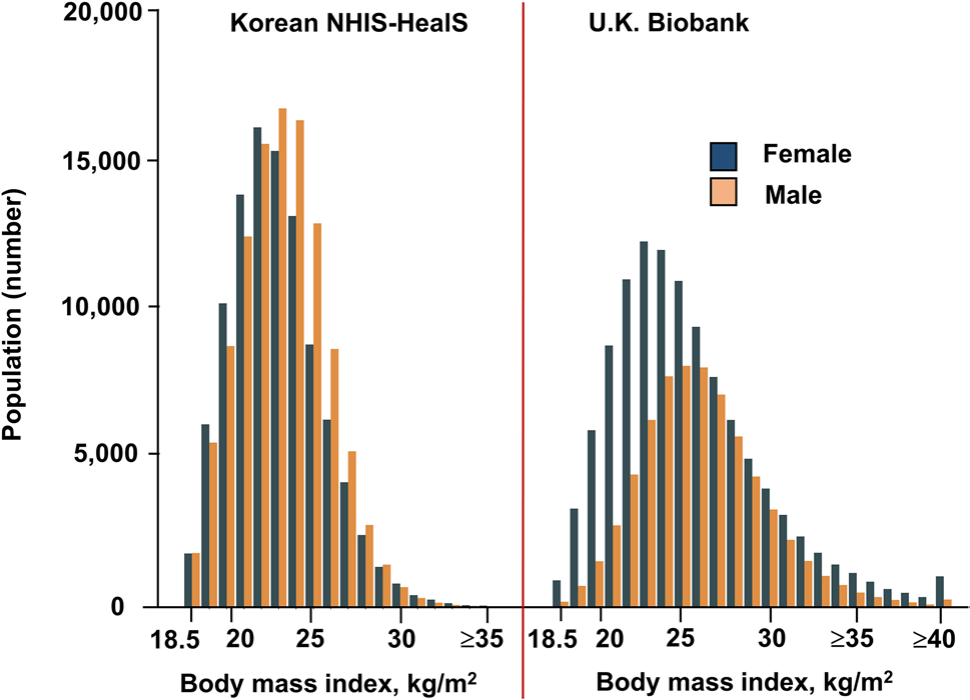
The distribution of participants in the Korean NHIS-HealS and U.K. Biobank cohorts. The cumulative incidence of new-onset AF was higher in the obese and overweight groups than in the normal-weight and underweight groups in both cohorts. *NHIS-HealS* National Health Insurance Service’s Health Screening.

Table 1 shows a comparison of baseline characteristics of participants from the Korean NHIS-HealS and U.K. Biobank cohorts. Among 401,206 participants from the Korean NHIS-HealS cohort (mean age 53.9 years; 54.9% men), 7228 (1.8%) were underweight, 253,522 (63.2%) had normal weights, 129,379 (32.2%) were overweight, and 11,077 (2.8%) were obese. Among 477,926 participants from the U.K. Biobank (mean age 56.6 years; 45.0% men), 2533 (0.5%) were underweight, 156,957 (32.8%) had normal weights, 202,488 (42.4%) were overweight, and 115,948 (24.3%) were obese. The proportions of obese (*P* < 0.001) and severely obese (BMI ≥ 40.0) (*P* < 0.001) individuals were higher in the U.K. Biobank cohort than the Korean NHIS-HealS cohort. This trend was consistently observed across both sexes (Fig. 2). The proportion of participants with abdominal obesity was smaller in the Korean NHIS-HealS cohort compared to the U.K. Biobank cohort (31.6% vs. 66.9%, *P* < 0.001).

**Table 1:** Baseline characteristics of the total population of the Korean NHIS-HealS cohorts and U.K. Biobank.

	Korean NHIS-HealS (n = 401,206)	U.K. Biobank (n = 477,926)	*P* value
Age, years	53.9 ± 7.2	56.6 ± 8.0	< 0.001
Male	220,318 (54.9)	214,850 (45.0)	< 0.001
BMI, kg/ m^2^	24.1 ± 2.9	27.4 ± 4.8	< 0.001
BMI categories			< 0.001
Underweight (< 18.5 kg/ m^2^)	7228 (1.8)	2533 (0.5)	
Normal (18.5 to < 25.0 kg/ m^2^)	253,522 (63.2)	156,957 (32.8)	
Overweight (25.0 to < 30.0 kg/ m^2^)	129,379 (32.2)	202,488 (42.4)	
Obese (30.0 to < 40.0 kg/ m^2^)	11,009 (2.7)	106,771 (22.3)	
Severely obese (≥ 40.0 kg/ m^2^)	68 (0.0)	9,177 (1.9)	
WC, cm	82.1 ± 7.8	90.1 ± 13.4	< 0.001
Abdominal obesity	121,006 (31.6)	319,774 (66.9)	< 0.001
SBP, mmHg	125.3 ± 16.3	137.8 ± 18.7	< 0.001
DBP, mmHg	78.4 ± 10.7	82.3 ± 10.2	< 0.001
Smoking	74,579 (19.6)	113,378 (25.8)	< 0.001
Alcohol^a^	107,119 (26.7)	22,824 (16.4)	< 0.001
Heart failure	9019 (2.2)	12,971 (2.7)	< 0.001
Hypertension	112,682 (28.1)	58,727 (12.3)	< 0.001
Diabetes mellitus	29,907 (7.5)	23,777 (5.0)	< 0.001
Ischemic stroke or TIA	14,633 (3.6)	7848 (1.6)	< 0.001
Previous MI	3410 (0.8)	821 (0.2)	< 0.001
Hyperthyroidism	10,030 (2.5)	4753 (1.0)	< 0.001
Hypothyroidism	10,335 (2.6)	25,493 (5.3)	< 0.001
Osteoporosis	49,382 (12.3)	9789 (2.0)	< 0.001
Dyslipidemia	95,543 (23.8)	65,651 (13.7)	< 0.001
ESRD or CKD	2747 (0.7)	4911 (1.0)	< 0.001
COPD	8138 (2.0)	2668 (0.6)	< 0.001
History of liver disease	83,625 (20.8)	1587 (0.3)	< 0.001
History of malignant neoplasm	24,711 (6.2)	42,414 (8.9)	< 0.001
Glucose, mg/dL	98.4 ± 26.9	92.2 ± 22.1	< 0.001
Creatinine, mg/dL	1.0 ± 1.0	0.8 ± 0.2	< 0.001
Cholesterol, mg/dL	198.9 ± 36.9	220.4 ± 43.9	< 0.001
Triglycerides, mg/dL	144.7 ± 98.3	154.5 ± 91.1	< 0.001
LDL, mg/dL	119.0 ± 38.1	137.8 ± 33.4	< 0.001
HDL, mg/dL	55.5 ± 29.6	56.0 ± 14.7	0.006

Values are presented as mean ± standard deviation or number (%).

*BMI* body mass index, *CKD* chronic kidney disease, *DBP* diastolic blood pressure, *ESRD* end-stage renal disease, *HDL* high-density lipoprotein, *LDL* low-density lipoprotein, *MI* myocardial infarction, *NHIS-HealS* National Health Insurance Service’s Health Screening, *SBP* systolic blood pressure, *TIA* transient ischemic attack, *WC* waist circumference.

^a^Alcohol intake was collected in the form of a survey and alcohol use was defined as alcohol use at least once a week.

The baseline characteristics for different BMI and WC categories are presented in Supplementary Tables S3–S5, respectively.

### AF and obesity

**Figure 3:**
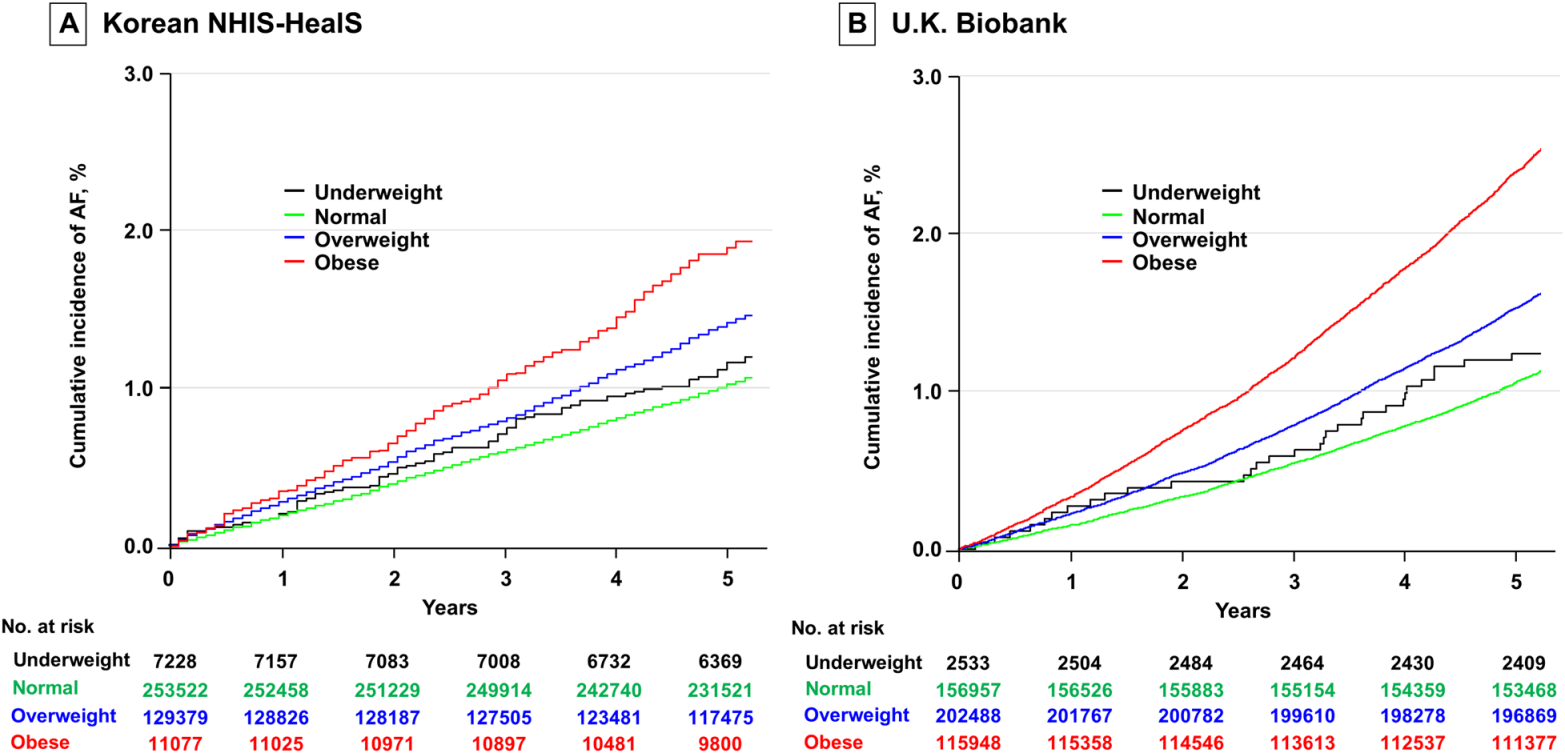
The cumulative incidence of AF across categories of body mass index in the Korean NHIS-HealS (**A**) and U.K. Biobank (**B**) cohorts. *AF* atrial fibrillation, *NHIS-HealS* National Health Insurance Service’s Health Screening.

In the Korean NHIS-HealS cohort, 8842 (2.2%) participants (62.8% male) developed AF during a mean follow-up period of 7.3 ± 1.5 years. The crude and adjusted incidences of AF by BMI category in the Korean NHIS-HealS cohort are presented in Table 2. The age- and sex-adjusted AF incidence rates were 3.10, 2.77, 3.52, and 4.97 per 1000 person-years in the underweight, normal-weight, overweight, and obese groups, respectively. The age- and sex-adjusted incidence rate of AF showed an increase in overweight and obese individuals across both sexes. The cumulative incidence of new-onset AF was higher in the obese and overweight groups than the normal-weight and underweight groups (Fig. 3A, P < 0.001).

**Table 2:** Age- and sex-adjusted incidence of AF by BMI in the Korean NHIS-HealS Cohort and the U.K. Biobank Cohort.

	Korean NHIS-HealS			
	Underweight (n = 7228)	Normal-weight (n = 253,522)	Overweight (n = 129,379)	Obese (n = 11,077)
Total				
Numbers of events/PYRs	168/51,441	4959/1,846,252	3340/936,372	375/78,471
AF incidence (/1000 PYRs)^a^	3.10	2.77	3.52	4.97
Female				
Numbers of events/PYRs	63/24,029	1821/853,713	1207/387,031	195/44,625
AF incidence (/1000 PYRs)^b^	2.67	2.26	2.88	4.08
Male				
Numbers of events/ PYRs	105/27,412	3138/992,539	2133/549,341	180/33,845
AF incidence (/1000 PYRs)^b^	3.45	3.19	4.05	5.71

	U.K. Biobank			
	Underweight	Normal-weight	Overweight	Obese
	(n = 2533)	(n = 156,957)	(n = 202,488)	(n = 115,948)
Overall				
Numbers of events/PYRs	80/28,376	5201/1,813,555	9706/2,320,859	8536/1,306,745
AF incidence (/1000 PYRs)^a^	4.22	3.35	3.95	6.54
Female				
Numbers of events/ PYRs	53/23,055	2481/1,198,874	3170/1,116,948	3292/707,559
AF incidence (/1000 PYRs)^b^	2.61	2.27	2.71	4.6
Male				
Numbers of events/ PYRs	27/5322	2720/614,681	6536/1,203,911	5244/599,186
AF incidence (/1000 PYRs)^b^	6.19	4.66	5.47	8.93

*NHIS-HealS* National Health Insurance Service’s Health Screening, *PYRs* person-years.

^a^Sex- and age-adjusted AF incidence.

^b^Age-adjusted AF incidence.

In the U.K. Biobank cohort, 23,523 (4.9%) participants (61.8% male) developed AF during a mean follow-up period of 11.4 ± 2.0 years. The overall age- and sex-adjusted AF incidence was significantly higher in the U.K. Biobank cohort than the Korean NHIS-HealS cohort (4.30 vs. 3.04 per 1000 person-years, *P* < 0.001). In the U.K. Biobank cohort, the overall age- and sex-adjusted AF incidence rates were 4.22, 3.35, 3.95, and 6.54 per 1000 person-years in the underweight, normal-weight, overweight, and obese groups, respectively (Table 2).

The age- and sex-adjusted incidence of AF increased in overweight and obese individuals across both sexes. The age- and sex-adjusted AF incidence in obese individuals was significantly higher in the U.K. Biobank cohort than the Korean NHIS-HealS cohort (*P* < 0.001). Across both sexes, the age-adjusted incidence of AF among obese individuals was significantly higher in the U.K. Biobank cohort than the Korean NHIS-HealS cohort (*P* < 0.001). The cumulative incidence of new-onset AF was greater in the obese and overweight groups than the normal-weight and underweight groups (Fig. 3B in the Supplement, *P* < 0.001).

### New-onset AF according to BMI category

After adjustment for clinical variables and competing risk of death, each 1-SD (Korea, 2.9 kg/m^2^; U.K., 4.8 kg/m^2^) increase in BMI correlated with a 8% (*P* < 0.001) or 28% (*P* < 0.001) greater risk of AF in the Korean NHIS-HealS cohort or U.K. Biobank cohort, respectively (Table 3). Regressions with four BMI categories (underweight, normal-weight, overweight, and obese) were evaluated to investigate the influence of BMI degree. After adjustment for clinical variables and competing risk of death, the risk of AF was increased in obese individuals (sHR, 1.41; 95% CI 1.26–1.58 [Korea] vs. sHR, 1.68; 95% CI 1.54–1.82 [U.K.]) (*P* = 0 0.032 for interaction).

**Table 3:** AF risk of BMI according to the age-, sex-, and clinical variable–adjusted model with competing risk of death in the total population.

	Korean NHIS-HealS		U.K. Biobank		*P* for interaction
	sHR (95% CI)	*P* value	sHR (95% CI)	*P* value	
With BMI as a 1-SD increase	1-SD (2.9 kg/ m^2^)		1-SD (4.8 kg/ m^2^)		
Age, sex-adjusted	1.16 (1.14–1.18)	< 0.001	1.36 (1.34–1.38)	< 0.001	< 0.001
Adjusted for clinical variables*	1.08 (1.06–1.11)	< 0.001	1.28 (1.23–1.32)	< 0.001	< 0.001
With BMI as a 1 kg/ m^2^ increase					
Age, sex-adjusted	1.05 (1.05–1.06)	< 0.001	1.07 (1.07–1.07)	< 0.001	< 0.001
Adjusted for clinical variables*	1.03 (1.02–1.04)	< 0.001	1.05 (1.05–1.06)	< 0.001	< 0.001
With BMI as a categorical variable					
Age, sex-adjusted					
Underweight (< 18.5 kg/ m^2^)	0.86 (0.73–1.01)	0.063	1.07 (0.86–1.32)	0.566	0.357
Normal (18.5 to < 25 kg/ m^2^)	1 (reference)		1 (reference)		
Overweight (25 to < 30 kg/ m^2^)	1.19 (1.10–1.28)	< 0.001	1.18 (1.14–1.21)	< 0.001	< 0.001
Obese (≥ 30 kg/ m^2^)	1.45 (1.19–1.77)	< 0.001	1.94 (1.87–2.00)	< 0.001	< 0.001
Adjusted for clinical variables*					
Underweight (< 18.5 kg/ m^2^)	1.18 (1.01–1.38)	0.043	0.86 (0.48–1.55)	0.619	0.309
Normal (18.5 to < 25 kg/ m^2^)	1 (reference)		1 (reference)		
Overweight (25 to < 30 kg/ m^2^)	1.15 (1.10–1.21)	< 0.001	1.10 (1.02–1.19)	0.011	0.001
Obese (≥ 30 kg/ m^2^)	1.41 (1.26–1.58)	< 0.001	1.68 (1.54–1.82)	< 0.001	0.032

*NHIS-HealS* National Health Insurance Service’s Health Screening, *sHR* subdistribution hazard ratio.

*Adjusted for age, sex, and clinical variables including smoking, alcohol use, heart failure, hypertension, diabetes mellitus, ischemic stroke or transient ischemic attack, previous myocardial infarction, hyperthyroidism, hypothyroidism, osteoporosis, dyslipidemia, end stage renal disease or chronic kidney disease, chronic obstructive pulmonary disorder, history liver disease, and history malignant neoplasm.

**Figure 4:**
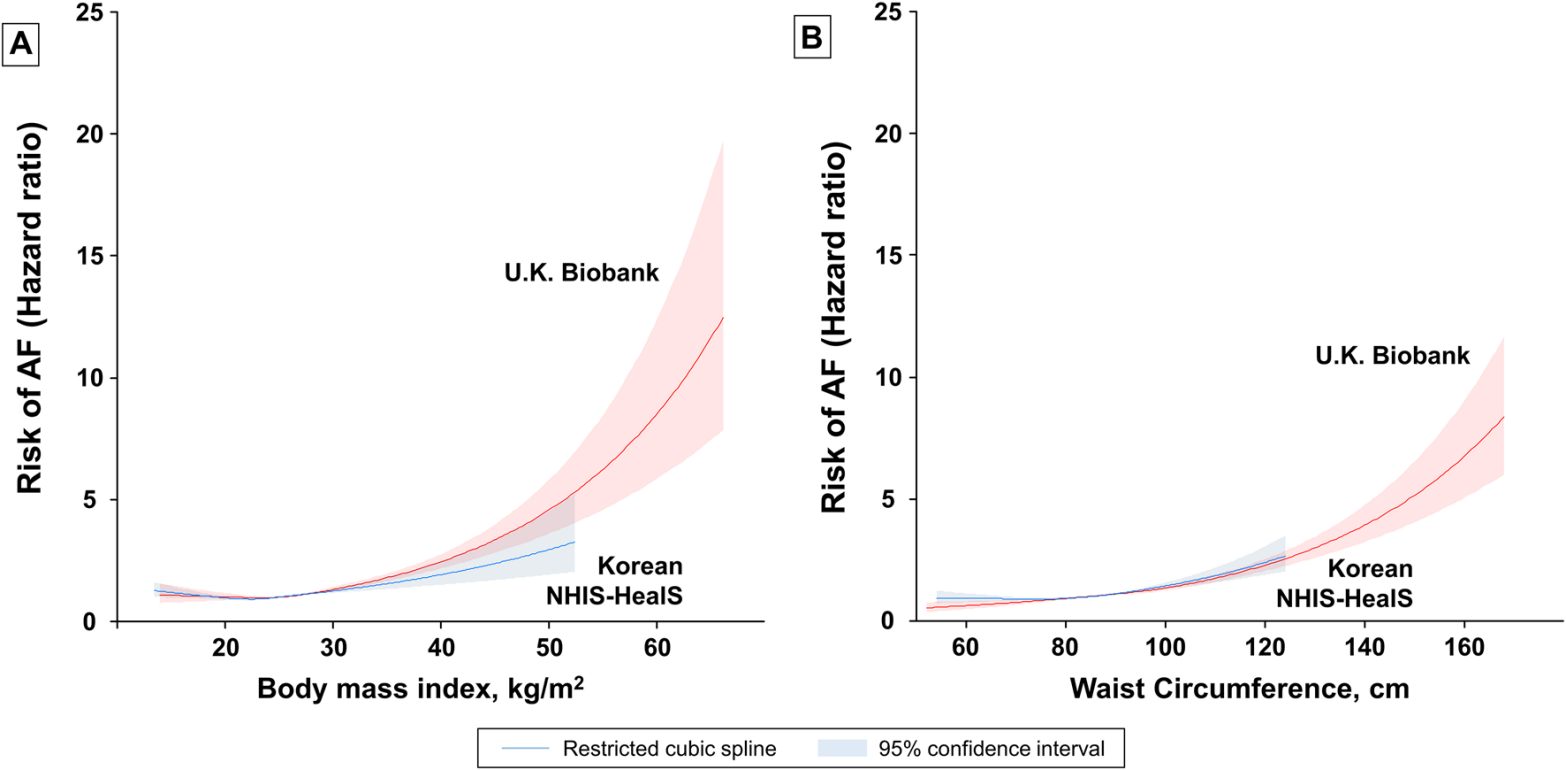
Spline curve of AF risk according to Body Mass Index (**A**) and waist circumference (**B**). (**A**) The trend of a rise in AF risk with increasing BMI was more prominent in the U.K. than Korea. (**B**) The trend of a rise in AF risk with increasing WC was more prominent in the U.K. than Korea. *AF* atrial fibrillation, *NHIS-HealS* National Health Insurance Service’s Health Screening.

In Fig. 4A, we can see spline curves between BMI and the HR of new-onset AF. Of note, there is a non-linear J-shaped association between continuous BMI variables and AF risk in both cohorts. The trend of a rise in AF risk with increasing BMI was more prominent in the U.K. than Korea.

### New-onset AF according to abdominal obesity

In individuals without and with abdominal obesity, the age- and sex-adjusted AF incidence rates in the Korean NHIS-HealS cohort were 2.64 and 3.72 per 1000 person-years and those of the U.K. Biobank cohort were 3.13 and 4.92 per 1000 person-years, respectively (Supplementary Table S8). The incidence of AF in individuals with abdominal obesity was significantly higher in the U.K. Biobank cohort than the Korean NHIS-HealS cohort (*P* < 0.001). Patients with abdominal obesity had a greater cumulative incidence of new-onset AF than those without abdominal obesity in both cohorts (log-rank *P* < 0.001) (Supplementary Figure S1). After adjustment for clinical variables and competing risk of death, each 1-SD (Korea, 7.8 cm; U.K., 13.4 cm) increase in WC was associated with an increase in the risk of AF risk by 12% (*P* < 0.001) and 37% (*P* < 0.001), respectively, in the Korean NHIS-HealS and U.K. Biobank cohorts, while a 1-SD increase in abdominal obesity was associated with an increase in the risk of AF risk by 20% (*P* < 0.001) and 42% (*P* < 0.001) (Supplementary Table S9).

Spline curves between WC and the risk of new-onset AF are presented in Fig. 4B. A linear association was found between continuous WC variables and the risk of AF in both cohorts.

### Sensitivity analysis

The age- and sex-adjusted incidence rates of AF in participants < 40 years and ≥ 70 years, are presented in Supplementary Table S10. The adjusted risk of AF in the first excluded participation is presented in Supplementary Table S11. The incidence and risk of AF increased with higher BMI. The age- and sex-adjusted incidence rates and adjusted risk of AF were significantly higher in the U.K. Biobank cohort than the Korean NHIS-HealS cohort.

## Discussion

The principal finding of this analysis is that overweightness and obesity are associated with an increase in age- and sex-adjusted AF incidence rates in both Korea and U.K. cohorts. The risk of incident AF continuously increased with higher BMI and WC values. Second, the overall incidence of incident AF was consistently higher in individuals from the U.K. Biobank cohort than the Korean NHIS-HealS cohort, irrespective of BMI and WC values. Third, there were small numbers of severely obese individuals in the Korean NHIS-HealS cohort, but, in the U.K. Biobank cohort, the incidence of AF continuously increased with higher BMI and WC values, even into the severely obese range. As far as we are aware, this is the first study to investigate whether obesity mediates the association with AF in Europe and Asia using patient-level data comparisons of two large cohort studies.

### Ethnic differences in AF

Epidemiological studies suggest that AF is more prevalent in whites than Asians/Afro-Caribbeans/other races^7–9,27^. Consistently, this study shows that the age- and sex-adjusted AF incidence rate was significantly higher in participants from the U.K. Biobank cohort than the Korean NHIS-HealS cohort (4.56 vs. 3.15 per 1000 person-years). Investigators from the Multi-Ethnic Study of Atherosclerosis (MESA) study estimated the age- and sex-adjusted incidence rates of hospitalized AF per 1000 person-years as 11.2 in whites, 5.8 in blacks, and 3.9 in Asians^7^. In patients with cardiac implantable electronic devices, the incidence of AF was 12.2 per 100 Afro-Caribbean beneficiaries per year compared to 17.6 per 100 non-black beneficiaries per year^8^.

The prevalence of AF was also significantly higher in whites than Asians. In a large survey of male veterans, the age-adjusted prevalence of AF was noted to be 3.6% in Asians and 5.7% in whites^27^. Interestingly, the lifetime risk of AF was also higher in whites than non-whites. In the Atherosclerosis Risk in Communities (ARIC) cohort, for example, the lifetime risk of AF was 36% in white male participants, 30% in white female participants, 21% in African American male participants, and 22% in African American female participants^28^.

### Obesity and new-onset AF risk

Although the precise mechanisms of racial differences in AF were not identified in this study, our investigation does show that obesity might be an important reason for the higher incidence of AF in the U.K. compared to Korea. Overweight and obese individuals are more prevalent in Western populations than Asian populations. Indeed, the prevalence of obesity (BMI > 30 kg/ m^2^) was almost 10 times higher in the U.K. than in Korea (2.7% vs. 24.3%) in this study. In a range of community-based cohorts, general obesity and increased BMI have been associated with an increased AF risk during follow-ups^29–31^. Particularly, considering that BMI was much less closely associated with AF when left atrial size was adjusted by the Framingham study, the presence of dilated left atria in obese individuals seems to be important in mechanism^30^.

Obesity and age are important predisposing factors to atrial fibrillation (AF)^32^. Age was observed as the greatest risk factor for AF compared to other risk factors. Physical changes such as body mass index decrease, alternation of muscles and fatty tissue composition accompany the aging process. The effect of obesity in the elderly is much more complex. Thus, increased risk of AF from higher BMI was most pronounced in the younger age group than the elderly^33^. Therefore, there is a risk of increasing the confounding effect by including patients over 70 into this study.

Considering that obesity is one of a limited number of modifiable risk factors for AF, it is therefore important to control obesity to prevent the pandemic of AF. We can visualize attention paid to obesity and other lifestyle factors as part of the characterization and integrated approach to AF care^34,35^ whereby adherence has been associated with improved clinical outcomes^36^ and recommended in international guidelines^37,38^. Visceral adiposity is associated with incident cardiovascular disease after adjustment for clinical risk factors and generalized adiposity^39^. However, previous studies did not report an additional risk of AF brought on by abdominal obesity beyond BMI. Also, we found a tendency for Asians to have more abdominal fat at lower BMIs than Europeans^40^. Indeed, the prevalence of overweightness and obesity in the Asian population is lower than that in the Western population, and amounts of abdominal fat at lower BMIs tend to be higher in Asians than Europeans^40^.

### Strengths and limitations of this study

The major strengths of this study are its large sample population drawn from two well-established nationwide cohorts in Korea and the U.K. and individual participant-level data comparisons. The large sample size also allowed us to perform joint and stratified analyses with sufficient statistical power. We also conducted a series of sensitivity analyses to show the robustness of the findings.

Yet, this study is subject to several limitations. First, we acknowledge there is a potential selection bias. The study subjects are slightly younger than the general population of Korea. Participants in the U.K. biobank were less likely to be obese, to smoke, and to drink alcohol than the general population, and self-reported health conditions were also fewer. Indeed, ≥ 1 health examinations in two years is not a mandatory requirement but instead a recommendation by the NHIS. In contrast, the U.K. Biobank consists of participants invited to participate in a research project with detailed screening and investigations. Second, the follow-up duration was relatively short, and those who died during the study period might have had serious diseases at baseline. Third, studies utilizing administrative databases (ICD-10 codes) might be susceptible to errors arising from coding inaccuracies. To minimize this problem, we applied the definition that has been previously validated in previous studies using the Korean NHIS sample cohort and UK biobank. Fourth, the incidence of AF may be underestimated in the present study because paroxysmal AF is frequently overlooked in clinical settings. Finally, there are several uncontrollable factors affecting the results, such as the time of enrolling participants, awareness of obesity and AF in both medical practitioners and patients, cultural differences, differences in socioeconomic factors (health insurance, education, etc.). Although we controlled for key personal characteristics and comorbidities, residual confounding was still possible and causal inferences cannot be made because of the nature of observational studies.

## Conclusion

Based on individual patient-level data from two large prospective nationwide Korea and U.K. cohort studies, we found that obesity was associated with AF in both populations, whereby the risk of incident AF continuously increased with increasing BMI and WC values, with a great risk seen in the U.K. cohort. Subjects in the U.K. cohort had a higher incidence of AF related to the high proportion of obese individuals, but the obesity-related risks of AF were also greater for the obese groups.

## Supplementary Information

Supplementary Information.

## Data Availability

The data presented in the study are available from NHIS and U.K. biobank for a fee via their data access procedure: https://www.ukbiobank.ac.uk/enable-your-research/register, https://opendata.hira.or.kr/or/orb/useGdInfo.do.

## Abbreviations

AF: Atrial fibrillation
BMI: Body mass index
CKD: Chronic kidney disease
ICD: International Classifications of Diseases
IRB: Institutional review board
MI: Myocardial infarction
NHIS-HealS: National Health Insurance Service’s Health Screening
SD: Standard deviation
WC: Waist circumference

## References

[1] Kim D (2018). 10-year nationwide trends of the incidence, prevalence, and adverse outcomes of non-valvular atrial fibrillation nationwide health insurance data covering the entire Korean population. Am. Heart J..

[2] Kim D (2018). Increasing trends in hospital care burden of atrial fibrillation in Korea, 2006 through 2015. Heart.

[3] Kim D (2021). Treatment timing and the effects of rhythm control strategy in patients with atrial fibrillation: Nationwide cohort study. BMJ.

[4] Joung B (2018). 2018 Korean guideline of atrial fibrillation management. Korean Circ. J..

[5] Burdett P, Lip GYH (2022). Atrial fibrillation in the UK: Predicting costs of an emerging epidemic recognizing and forecasting the cost drivers of atrial fibrillation-related costs. Eur. Heart J. Qual. Care Clin. Outcomes.

[6] Zhang J, Johnsen SP, Guo Y, Lip GY (2021). Epidemiology of atrial fibrillation: Geographic/ecological risk factors, age, sex, genetics. Cardiac. Electrophysiol. Clin..

[7] Rodriguez CJ (2015). Atrial fibrillation incidence and risk factors in relation to race-ethnicity and the population attributable fraction of atrial fibrillation risk factors: The Multi-Ethnic Study of Atherosclerosis. Ann. Epidemiol..

[8] Chen ML (2019). Risk of atrial fibrillation in black versus white medicare beneficiaries with implanted cardiac devices. J. Am. Heart Assoc..

[9] Piccini JP (2012). Incidence and prevalence of atrial fibrillation and associated mortality among Medicare beneficiaries, 1993–2007. Circ. Cardiovasc. Qual. Outcomes.

[10] Heeringa J (2006). Prevalence, incidence and lifetime risk of atrial fibrillation: The Rotterdam study. Eur. Heart J..

[11] Vermond RA (2015). Incidence of atrial fibrillation and relationship with cardiovascular events, heart failure, and mortality: A community-based study from the Netherlands. J. Am. Coll. Cardiol..

[12] Benjamin EJ (1994). Independent risk factors for atrial fibrillation in a population-based cohort .The Framingham Heart Study. JAMA J. Am. Med. Assoc..

[13] Miyasaka Y (2006). Secular trends in incidence of atrial fibrillation in Olmsted County, Minnesota, 1980 to 2000, and implications on the projections for future prevalence. Circulation.

[14] Rahman F, Kwan GF, Benjamin EJ (2014). Global epidemiology of atrial fibrillation. Nat. Rev. Cardiol..

[15] Schnabel R, Yin X, Gona P (2014). Fifty-year trends in atrial fibrillation prevalence, incidence, risk factors, and mortality in the community. Lancet.

[16] Pathak RK (2014). Aggressive risk factor reduction study for atrial fibrillation and implications for the outcome of ablation: The ARREST-AF cohort study. J. Am. Coll. Cardiol..

[17] Baek YS (2017). Associations of abdominal obesity and new-onset atrial fibrillation in the general population. J. Am. Heart Assoc..

[18] Rankinen T, Kim SY, Perusse L, Despres JP, Bouchard C (1999). The prediction of abdominal visceral fat level from body composition and anthropometry: ROC analysis. Int. J. Obes. Relat. Metab. Disord..

[19] Lim YM (2019). Body mass index variability and long-term risk of new-onset atrial fibrillation in the general population: A Korean nationwide cohort study. Mayo Clin. Proc..

[20] Seong SC (2017). Cohort profile: The national health insurance service-national health screening cohort (NHIS-HEALS) in Korea. BMJ Open.

[21] Fry A (2017). Comparison of sociodemographic and health-related characteristics of UK Biobank participants with those of the general population. Am. J. Epidemiol..

[22] Sudlow C (2015). UK biobank: An open access resource for identifying the causes of a wide range of complex diseases of middle and old age. PLoS Med..

[23] Kim D (2019). Risk of dementia in stroke-free patients diagnosed with atrial fibrillation: Data from a population-based cohort. Eur. Heart J..

[24] Kim TH (2019). Effect of hypertension duration and blood pressure level on ischaemic stroke risk in atrial fibrillation: Nationwide data covering the entire Korean population. Eur. Heart J..

[25] Alberti KG, Zimmet P, Shaw J (2006). Metabolic syndrome—a new world-wide definition. A Consensus Statement from the International Diabetes Federation. Diabet. Med..

[26] Fine JP, Gray RJ (1999). A proportional hazards model for the subdistribution of a competing risk. J. Am. Stat. Assoc..

[27] Borzecki AM (2008). Racial differences in the prevalence of atrial fibrillation among males. J. Natl. Med. Assoc..

[28] Alonso A (2009). Incidence of atrial fibrillation in whites and African-Americans: The Atherosclerosis Risk in Communities (ARIC) study. Am. Heart J..

[29] Murphy NF (2006). Long-term cardiovascular consequences of obesity: 20-year follow-up of more than 15 000 middle-aged men and women (the Renfrew–Paisley study). Eur. Heart J..

[30] Wang TJ (2004). Obesity and the risk of new-onset atrial fibrillation. J. Am. Med. Assoc..

[31] Tedrow UB (2010). The long- and short-term impact of elevated body mass index on the risk of new atrial fibrillation the WHS (Women's Health Study). J. Am. Coll. Cardiol..

[32] Staerk L, Sherer JA, Ko D, Benjamin EJ, Helm RH (2017). Atrial fibrillation: Epidemiology, pathophysiology, and clinical outcomes. Circ. Res..

[33] Choi E-K (2022). Associations between obesity parameters and the risk of incident atrial fibrillation and ischaemic stroke in different age groups. Front. Cardiovasc. Med..

[34] Lip GYH (2017). The ABC pathway: An integrated approach to improve AF management. Nat. Rev. Cardiol..

[35] Potpara TS (2021). The 4S-AF scheme (stroke risk; symptoms; severity of burden; substrate): A novel approach to in-depth characterization (rather than classification) of atrial fibrillation. Thromb. Haemost..

[36] Romiti GF (2022). Adherence to the 'atrial fibrillation better care' pathway in patients with atrial fibrillation: Impact on clinical outcomes—a systematic review and meta-analysis of 285,000 patients. Thromb. Haemost..

[37] Chao TF (2022). 2021 Focused update consensus guidelines of the Asia Pacific heart rhythm Society on Stroke Prevention in Atrial Fibrillation: Executive summary. Thromb. Haemost..

[38] Hindricks G (2021). 2020 ESC Guidelines for the diagnosis and management of atrial fibrillation developed in collaboration with the European Association for Cardio-Thoracic Surgery (EACTS): The Task Force for the diagnosis and management of atrial fibrillation of the European Society of Cardiology (ESC) Developed with the special contribution of the European Heart Rhythm Association (EHRA) of the ESC. Eur. Heart J..

[39] Britton KA (2013). Body fat distribution, incident cardiovascular disease, cancer, and all-cause mortality. J. Am. Coll. Cardiol..

[40] Consultation WHOE (2004). Appropriate body-mass index for Asian populations and its implications for policy and intervention strategies. Lancet.

