# Supplementary material for: Association of obesity with incident atrial fibrillation in Korea and the United Kingdom
Source: Sci Rep. 2023 Mar 30;13:5197. doi: 10.1038/s41598-023-32229-9 (PMC10063613; doi:10.1038/s41598-023-32229-9)

**Supplementary Materials**

**Supplementary Table S1. Definitions Used for Defining the Comorbidities.**

| **Comorbidities** | **Korea NHIS** | | **UK Biobank** | |
| --- | --- | --- | --- | --- |
|  | **Definitions** | **Used codes or conditions** | **Definitions** | **Used codes or conditions** |
| Atrial fibrillation | Defined from diagnosis^a^ | ICD-10: I48 | Defined from UK Biobank self-report or diagnosis^a^ | Self-reported non-cancer illness code: 1471, 1483  ICD-10: I48 |
| Ischemic stroke | Defined from diagnosis^a^ | ICD-10: I63, I64 | Defined from UK Biobank self-report or diagnosis^a^ | Self-reported non-cancer illness code: 1583  ICD-10: I63, I64 |
| TIA | Defined from diagnosis^a^ | ICD-10: G45 | Defined from UK Biobank self-report or diagnosis^a^ | Self-reported non-cancer illness code: 1082  ICD-10: G45 |
| Hemorrhagic stroke | Defined from diagnosis^a^ | ICD-10: I60, I61, I62 | Defined from UK Biobank self-report or diagnosis^a^ | Self-reported non-cancer illness code: 1086, 1491  ICD-10: I60, I61, I62 |
| Heart failure | Defined from diagnosis^a^ | ICD-10: I11.0, I50, I97.1 | Defined from UK Biobank self-report or diagnosis^a^ | Self-reported non-cancer illness code: 1076  ICD-10: I11.0, I50, I97.1 |
| Diabetes mellitus | Defined from diagnosis^a^ plus treatment | ICD-10: E10, E11, E12, E13, E14  Treatment: all kinds of oral antidiabetics and insulin | Defined from UK Biobank self-report or diagnosis^a^ | Self-reported non-cancer illness code: 1220, 1222, 1223, 1521  ICD-10: E10, E11, E12, E13, E14 |
| Hypertension | Defined from diagnosis^a^ plus treatment | ICD-10: I10, I11, I12, I13, I15  Treatment: all kinds of blood pressure lowering medications (>1 month). | Defined from UK Biobank self-report or diagnosis^a^ | Self-reported non-cancer illness code: 1065, 1072  ICD-10: I10, I11, I12, I13, I15 |
| Previous myocardial infarction (MI) | Defined from diagnosis^a^ | ICD-10: I21, I22, I25.2 | Defined from UK Biobank self-report or diagnosis^a^ | Self-reported non-cancer illness code: 1075  ICD-10: I21, I22, I25.2 |
| Peripheral arterial disease | Defined from diagnosis^a^ | ICD-10: I70, I71 | Defined from UK Biobank self-report or diagnosis^a^ | Self-reported non-cancer illness code: 1067, 1087  ICD-10: I70, I71 |
| Dyslipidemia | Defined from diagnosis^a^ | ICD-10: E78 | Defined from UK Biobank self-report or diagnosis^a^ | Self-reported non-cancer illness code: 1473  ICD-10: E78 |
| COPD | Defined from diagnosis^a^ plus treatment | ICD-10: J42, J43(except J43.0), J44  Treatment: SABA, SAMA, LABA, LAMA, ICS, ICS+LABA, or methylxanthine (>1 month). | Defined from UK Biobank self-report or diagnosis^a^ | Self-reported non-cancer illness code: 1112, 1113, 1472  ICD-10: J42, J43(except J43.0), J44 |
| Chronic renal failure | Defined from eGFR (if laboratory value was not available, diagnosis code was used) | eGFR <60 mL/min per 1.73 m^2^  ICD-10: N18, N19 | Defined from eGFR (if laboratory value was not available, self-report or diagnosis code was used) | eGFR <60 mL/min per 1.73 m^2^  Self-reported non-cancer illness code:1192, 1194  ICD-10: N18, N19 |
| ESRD | Defined from national registry for severe illness or history of renal replacement therapy (hemodialysis, peritoneal dialysis, or kidney transplant) | Patients with ESRD undergoing chronic dialysis or received a kidney transplant. | Defined from UK Biobank self-report or procedure codes related to renal replacement therapy (hemodialysis, peritoneal dialysis, or kidney transplant) | Self-reported non-cancer illness code: 1193, 1195, 1580, 1581, 1582  Procedure codes: L74, M01, M02.3, M08.4, M17, X40, X41, X42 |
| Malignancy | Defined from diagnoses of cancer (non-benign) | ICD-10: C00-C97 | Defined from UK Biobank self-report or diagnoses of cancer (non-benign) | Self-reported cancer illness code: all  ICD-10: C00-C97 |
| Hypertrophic cardiomyopathy | Defined from diagnosis | ICD-10: I42.1, I42.2 | Defined from UK Biobank self-report or diagnosis | Self-reported non-cancer illness code: 1588  ICD-10: I42.1, I42.2 |
| Potential absence of non-valvular atrial fibrillation | Defined from any diagnoses of mitral stenosis or claim for heart valve surgery | ICD-10: I05.0, I05.2, I34.2, Z95.2-4  Claim for valve replacement or valvuloplasty: O1730, O1740, O1750, O1760, O1770, O1781, O1782, O1783, O1791, O1792, O1793, O1794, O1795, O1796, O1797, O1798 | Defined from UK Biobank self-report / any diagnoses of mitral stenosis or procedure codes for heart valve surgery | Self-reported non-cancer illness code: 1489  ICD-10: I05.0, I05.2, I34.2, Z95.2-4  Procedure codes: K25, K26, K27, K28, K29, K30, K31, K32, K33, K34, K35 |
| Hyperthyroidism | Defined from diagnosis^a^ | ICD-10: E05 | Defined from UK Biobank self-report or diagnosis^a^ | Self-reported non-cancer illness code: 1225, 1522  ICD-10: E05 |
| Hypothyroidism | Defined from diagnosis^a^ | ICD-10: E03 | Defined from UK Biobank self-report or diagnosis^a^ | Self-reported non-cancer illness code: 1226  ICD-10: E03 |
| Venous thrombosis including pulmonary embolism | Defined from diagnosis^a^ | ICD-10: I26, I80.1, I80.2, I80.3, I80.8, I80.9, I81, I82, I63.6, O22.3, O22.5, O87.1, O87.3, G80 | Defined from UK Biobank self-report or diagnosis^a^ | Self-reported non-cancer illness code: 1068, 1093, 1094  ICD-10: I26, I80.1, I80.2, I80.3, I80.8, I80.9, I81, I82, I63.6, O22.3, O22.5, O87.1, O87.3, G80 |

^a^ To ensure accuracy, comorbidities were established based on more than one hospital-inpatient or two outpatient (= primary care in United Kingdom) records of ICD-10 codes in the database.

**Supplementary Table S2. Definitions Used for Defining the Outcomes.**

| **Comorbidities** | **Korea NHIS** | | **UK Biobank** | |
| --- | --- | --- | --- | --- |
|  | **Definitions** | **Used codes or conditions** | **Definitions** | **Used codes or conditions** |
| New onset atrial fibrillation (AF) | Defined from diagnosis^a^ or related death without previous insurance claim for AF | ICD-10: I48 | Defined from diagnosis^a^ or related death without previous history of AF | ICD-10: I48 |
| All-cause death | Data related to death were confirmed at the National Population Register of the Korea National Statistical Office, where deaths are centrally registered based on the death certificate. | The cause of death was determined based on the ICD-10 code written on the death certificate. | Data related to death were confirmed by the death registries of United Kingdom | The cause of death was determined based on the ICD-10 code written on the death certificate. |

^a^ To ensure accuracy, comorbidities were established based on more than one hospital-inpatient or two outpatient (= primary care in United Kingdom) records of ICD-10 codes in the database.

**Supplementary Table S3. Baseline Characteristics by BMI of the Korean NHIS-HealS**

|  | **Underweight** | **Normal-weight** | **Overweight** | **Obese** | *P* value |
| --- | --- | --- | --- | --- | --- |
|  | **(n = 7,228)** | **(n = 253,522)** | **(n = 129,379)** | **(n = 11,077)** |  |
| Age, years | 54.8 ± 8.0 | 53.6 ± 7.2 | 54.3 ± 7.2 | 54.8 ± 7.2 | <0.001 |
| Male | 3,892 (53.8) | 135,931 (53.6) | 75,733 (58.5) | 4,762 (43.0) | <0.001 |
| BMI, kg/m^2^ | 17.6 ± 0.8 | 22.5 ± 1.6 | 26.7 ± 1.3 | 31.7 ± 2.0 | <0.001 |
| WC, cm | 69.6 ± 5.8 | 79.1 ± 6.3 | 87.4 ± 5.9 | 95.7 ± 7.1 | <0.001 |
| Abdominal obesity | 72 (1.1) | 36,643 (15.1) | 74,259 (60.1) | 10,032 (95.8) | <0.001 |
| SBP, mmHg | 118.4 ± 16.5 | 123.3 ± 16.0 | 128.9 ± 16.0 | 133.5 ± 16.7 | <0.001 |
| DBP, mmHg | 74.3 ± 10.7 | 77.1 ± 10.5 | 80.6 ± 10.6 | 83.2 ± 10.9 | <0.001 |
| Smoking | 1,972 (28.7) | 48,195 (20.0) | 22,894 (18.7) | 1,518 (14.5) | <0.001 |
| Alcohol | 1,729 (23.9) | 65,534 (25.8) | 37,323 (28.8) | 2,533 (22.9) | <0.001 |
| Heart failure | 83 (1.1) | 4231 (1.7) | 4019 (3.1) | 686 (6.2) | <0.001 |
| Hypertension | 1,034 (14.3) | 57,969 (22.9) | 47,722 (36.9) | 5,957 (53.8) | <0.001 |
| Diabetes mellitus | 347 (4.8) | 15,943 (6.3) | 12,014 (9.3) | 1,603 (14.5) | <0.001 |
| Ischemic stroke or TIA | 170 (2.4) | 7,957 (3.1) | 5,820 (4.5) | 686 (6.2) | <0.001 |
| Previous MI | 44 (0.6) | 1,815 (0.7) | 1,396 (1.1) | 155 (1.4) | <0.001 |
| Hyperthyroidism | 219 (3.0) | 6,416 (2.5) | 3,088 (2.4) | 307 (2.8) | <0.001 |
| Hypothyroidism | 212 (2.9) | 6,575 (2.6) | 3,186 (2.5) | 362 (3.3) | <0.001 |
| Osteoporosis | 968 (13.4) | 31,058 (12.3) | 15,596 (12.1) | 1,760 (15.9) | <0.001 |
| Dyslipidemia | 1,009 (14.0) | 52,542 (20.7) | 37,745 (29.2) | 4,247 (38.3) | <0.001 |
| ESRD or CKD | 56 (0.8) | 1,549 (0.6) | 1,027 (0.8) | 115 (1.0) | <0.001 |
| COPD | 293 (4.1) | 4,669 (1.8) | 2,785 (2.2) | 391 (3.5) | <0.001 |
| History of malignant neoplasm | 599 (8.3) | 15,723 (6.2) | 7,742 (6.0) | 647 (5.8) | <0.001 |
| Glucose, mmol/L | 5.24 ± 1.69 | 5.37 ± 1.44 | 5.61 ± 1.54 | 5.89 ± 1.76 | <0.001 |
| Creatinine, mg/dL | 1.0 ± 1.0 | 1.0 ± 1.0 | 1.0 ± 1.1 | 0.9 ± 0.8 | <0.001 |
| Cholesterol, mg/dL | 185.2 ± 35.2 | 196.6 ± 36.3 | 203.4 ± 37.4 | 207.1 ± 38.7 | 0.025 |
| Triglycerides, mg/dL | 110.0 ± 86.3 | 133.4 ± 90.9 | 163.5 ± 105.8 | 177.7 ± 114.4 | <0.001 |
| LDL, mg/dL | 107.9 ± 35.4 | 118.2 ± 38.1 | 120.9 ± 37.9 | 122.7 ± 39.3 | <0.001 |
| HDL, mg/dL | 59.8 ± 15.0 | 56.8 ± 29.0 | 53.4 ± 32.1 | 51.6 ± 17.4 | <0.001 |

Values are presented as mean ± standard deviation or number (%).

Abbreviations: BMI, Body mass index; CKD, chronic kidney disease; DBP, diastolic blood pressure; ESRD, end-stage renal disease; HDL, high-density lipoprotein; LDL, low-density lipoprotein; MI, myocardial infarction; NHIS-HealS; National Health Insurance Service’s-Health Screening; SBP, systolic blood pressure; TIA, transient ischemic attack; WC, waist circumference.

**Supplementary Table S4. Baseline Characteristics by BMI of the U.K. Biobank**

|  | **Underweight** | **Normal-weight** | **Overweight** | **Obese** | *P* value |
| --- | --- | --- | --- | --- | --- |
|  | **(n = 2,533)** | **(n = 156,957)** | **(n = 202,488)** | **(n = 115,948)** |  |
| Age, years | 55.6 ± 8.0 | 55.8 ± 8.1 | 57.1 ± 7.9 | 57.0 ± 7.8 | <0.001 |
| Male | 519 (20.5) | 54,032 (34.4) | 106,147 (52.4) | 54,152 (46.7) | <0.001 |
| BMI, kg/m^2^ | 17.6 ± 0.8 | 22.9 ± 1.5 | 27.3 ± 1.4 | 34.0 ± 3.9 | <0.001 |
| WC, cm | 66.5 ± 5.8 | 78.6 ± 8.1 | 90.9 ± 8.4 | 104.9 ± 11.0 | <0.001 |
| Abdominal obesity | 20 (0.8) | 37,160 (23.7) | 167,184 (82.6) | 115,410 (99.6) | <0.001 |
| SBP, mmHg | 126.2 ± 19.3 | 132.8 ± 18.7 | 139.3 ± 18.2 | 142.0 ± 17.8 | <0.001 |
| DBP, mmHg | 75.1 ± 10.3 | 78.7 ± 9.8 | 83.0 ± 9.8 | 86.0 ± 9.8 | <0.001 |
| Smoking | 319 (15.8) | 28,572 (20.0) | 50,821 (27.2) | 33,666 (31.3) | <0.001 |
| Alcohol | 52 (7.3) | 7,082 (13.2) | 10,683 (18.4) | 5,007 (18.8) | <0.001 |
| Heart failure | 54 (2.1) | 3,169 (2.0) | 5,177 (2.6) | 4,571 (3.9) | <0.001 |
| Hypertension | 131 (5.2) | 9,486 (6.0) | 23,889 (11.8) | 25,221 (21.8) | <0.001 |
| Diabetes mellitus | 32 (1.3) | 2,724 (1.7) | 8,215 (4.1) | 12,806 (11.0) | <0.001 |
| Ischemic stroke or TIA | 28 (1.1) | 1,756 (1.1) | 3,288 (1.6) | 2,776 (2.4) | <0.001 |
| Previous MI | 5 (0.2) | 185 (0.1) | 344 (0.2) | 287 (0.2) | <0.001 |
| Hyperthyroidism | 26 (1.0) | 1,655 (1.1) | 1,854 (0.9) | 1,218 (1.1) | <0.001 |
| Hypothyroidism | 106 (4.2) | 6,725 (4.3) | 9,650 (4.8) | 9,012 (7.8) | <0.001 |
| Osteoporosis | 214 (8.4) | 4,545 (2.9) | 3,392 (1.7) | 1,638 (1.4) | <0.001 |
| Dyslipidemia | 118 (4.7) | 12,523 (8.0) | 29,518 (14.6) | 23,492 (20.3) | <0.001 |
| ESRD or CKD | 29 (1.1) | 988 (0.6) | 1,948 (1.0) | 1,946 (1.7) | <0.001 |
| COPD | 47 (1.9) | 760 (0.5) | 1,008 (0.5) | 853 (0.7) | <0.001 |
| History of malignant neoplasm | 292 (11.5) | 14,597 (9.3) | 17,451 (8.6) | 10,074 (8.7) | <0.001 |
| Glucose, mmol/L | 4.89 ± 0.94 | 4.95 ± 0.93 | 5.09 ± 1.11 | 97.3 ± 5.40 | <0.001 |
| Creatinine, mg/dL | 0.7 ± 0.2 | 0.8 ± 0.2 | 0.8 ± 0.2 | 0.8 ± 0.2 | <0.001 |
| Cholesterol, mg/dL | 214.5 ± 40.9 | 220.3 ± 41.5 | 222.8 ± 44.4 | 216.7 ± 46.1 | 0.025 |
| Triglycerides, mg/dL | 95.3 ± 49.3 | 119.8 ± 65.5 | 162.2 ± 91.4 | 189.8 ± 103.5 | <0.001 |
| LDL, mg/dL | 125.4 ± 29.6 | 134.7 ± 31.7 | 140.5 ± 33.7 | 137.5 ± 34.8 | <0.001 |
| HDL, mg/dL | 70.1 ± 17.1 | 62.7 ± 15.2 | 54.5 ± 13.6 | 49.5 ± 12.0 | <0.001 |

Values are presented as mean ± standard deviation or number (%).

Abbreviations: BMI, Body mass index; CKD, chronic kidney disease; DBP, diastolic blood pressure; ESRD, end-stage renal disease; HDL, high-density lipoprotein; LDL, low-density lipoprotein; MI, myocardial infarction; NHIS-HealS; National Health Insurance Service’s-Health Screening; SBP, systolic blood pressure; TIA, transient ischemic attack; WC, waist circumference.

**Supplementary Table S5. Baseline Characteristics by Abdominal Obesity in the Korean NHIS-HealS Cohorts and U.K. Biobank.**

|  | **Korean NHIS-HealS** | | **U.K. Biobank** | |
| --- | --- | --- | --- | --- |
|  | **Abdominal obesity (-)** | **Abdominal obesity (+)** | **Abdominal obesity (-)** | **Abdominal obesity (+)** |
|  | **(n = 261,536)** | **(n = 121,006)** | **(n = 158,049)** | **(n = 319,774)** |
| Age, years | 53.0 ± 7.0 | 55.6 ± 7.2 | 55.3 ± 8.1 | 57.3 ± 7.8 |
| Male | 163137 (62.4) | 45419 (37.5) | 56099 (35.5) | 158710 (49.6) |
| BMI, kg/m^2^ | 23.0 ± 2.3 | 26.4 ± 2.6 | 23.3 ± 2.3 | 29.4 ± 4.4 |
| WC, cm | 78.9 ± 6.3 | 88.9 ± 6.1 | 76.8 ± 6.9 | 96.7 ± 10.8 |
| Abdominal obesity | 0 (0.0) | 121006 (100.0) | 0 (0.0) | 319774 (100.0) |
| SBP, mmHg | 123.6 ± 15.9 | 128.4 ± 16.4 | 132.9 ± 18.7 | 140.2 ± 18.2 |
| DBP, mmHg | 77.6 ± 10.6 | 79.9 ± 10.7 | 78.9 ± 9.8 | 84.0 ± 9.9 |
| Smoking | 54114 (21.9) | 15583 (13.5) | 27645 (19.1) | 85709 (29.1) |
| Alcohol | 75909 (29.0) | 25761 (21.3) | 7101 (13.3) | 15722 (18.3) |
| Heart failure | 3869 (1.5) | 4543 (3.8) | 2995 (1.9) | 9969 (3.1) |
| Hypertension | 57749 (22.1) | 48558 (40.1) | 9047 (5.7) | 49653 (15.5) |
| Diabetes mellitus | 15498 (5.9) | 12143 (10.0) | 2394 (1.5) | 21369 (6.7) |
| Ischemic stroke or TIA | 7370 (2.8) | 6283 (5.2) | 1620 (1.0) | 6227 (1.9) |
| Previous MI | 1882 (0.7) | 1260 (1.0) | 138 (0.1) | 682 (0.2) |
| Hyperthyroidism | 5949 (2.3) | 3687 (3.0) | 1554 (1.0) | 3198 (1.0) |
| Hypothyroidism | 5941 (2.3) | 4011 (3.3) | 6602 (4.2) | 18888 (5.9) |
| Osteoporosis | 24618 (9.4) | 22895 (18.9) | 4223 (2.7) | 5560 (1.7) |
| Dyslipidemia | 52458 (20.1) | 38824 (32.1) | 11843 (7.5) | 53786 (16.8) |
| ESRD or CKD | 1540 (0.6) | 1031 (0.9) | 958 (0.6) | 3948 (1.2) |
| COPD | 4223 (1.6) | 3338 (2.8) | 596 (0.4) | 2070 (0.6) |
| History of malignant neoplasm | 15553 (5.9) | 7609 (6.3) | 13961 (8.8) | 28430 (8.9) |
| Glucose, mmol/L | 5.38 ± 1.40 | 5.58 ± 1.56 | 4.93 ± 0.88 | 5.21 ± 1.35 |
| Creatinine, mg/dL | 1.0 ± 1.0 | 1.0 ± 1.1 | 0.8 ± 0.2 | 0.8 ± 0.2 |
| Cholesterol, mg/dL | 196.4 ± 35.9 | 204.4 ± 37.8 | 219.4 ± 41.0 | 221.0 ± 45.3 |
| Triglycerides, mg/dL | 135.7 ± 94.6 | 161.6 ± 102.8 | 116.6 ± 63.8 | 173.4 ± 96.6 |
| LDL, mg/dL | 117.4 ± 37.9 | 122.2 ± 38.3 | 134.0 ± 31.3 | 139.6 ± 34.3 |
| HDL, mg/dL | 56.4 ± 29.3 | 53.9 ± 30.1 | 62.8 ± 15.2 | 52.7 ± 13.3 |

Values are presented as mean ± standard deviation or number (%).

Abbreviations: BMI, Body mass index; CKD, chronic kidney disease; DBP, diastolic blood pressure; ESRD, end-stage renal disease; HDL, high-density lipoprotein; LDL, low-density lipoprotein; MI, myocardial infarction; NHIS-HealS; National Health Insurance Service-Health Screening; SBP, systolic blood pressure; TIA, transient ischemic attack; WC, waist circumference.

Abdominal obesity defined as WC ≥90 cm for men, ≥80 cm for women.

**Supplementary Table S6. Crude Incidence of AF by BMI in the Korean NHIS-HealS Cohort and the U.K. Biobank Cohort.**

|  | **Korean NHIS-HealS** | | | | | | |
| --- | --- | --- | --- | --- | --- | --- | --- |
|  | **Underweight** | **Normal-weight** | | **Overweight** | | **Obese** | |
|  | **(n = 7,228)** | **(n = 253,522)** | | **(n = 129,379)** | | **(n = 11,077)** | |
| ***Total*** |  |  | |  | |  | |
| Crude AF incidence^a^ |  |  | |  | |  | |
| 40–50 years | 1.77 | 1.39 | | 1.91 | | 3.01 | |
| 50–60 years | 2.43 | 2.3 | | 3.22 | | 4.06 | |
| 60–70 years | 5.86 | 5.34 | | 6.15 | | 7.6 | |
| ***Female*** |  |  | |  | |  | |
| Crude AF incidence |  |  | |  | |  | |
| 40–50 years | 1.32 | 1.12 | | 1.47 | | 2.96 | |
| 50–60 years | 1.98 | 1.83 | | 2.48 | | 3.42 | |
| 60–70 years | 5.11 | 4.11 | | 4.98 | | 6.25 | |
| ***Male*** |  |  | |  | |  | |
| Crude AF incidence |  |  | |  | |  | |
| 40–50 years | 2.23 | 1.6 | | 2.09 | | 3.04 | |
| 50–60 years | 2.81 | 2.73 | | 3.77 | | 4.95 | |
| 60–70 years | 6.42 | 6.45 | | 7.55 | | 11.14 | |
|  | **U.K. Biobank** | | | | | | |
|  | **Underweight** | **Normal-weight** | | **Overweight** | | **Obese** | |
|  | **(n = 2,533)** | **(n = 156,957)** | | **(n = 202,488)** | | **(n = 115,948)** | |
| ***Total*** |  |  | |  | |  | |
| Crude AF incidence^a^ |  |  | |  | |  | |
| 40–50 years | 0.38 | 0.6 | | 0.85 | | 1.61 | |
| 50–60 years | 1.63 | 1.74 | | 2.35 | | 4.26 | |
| 60–70 years | 6.02 | 5.48 | | 7.17 | | 10.93 | |
| ***Female*** |  |  | |  | |  | |
| Crude AF incidence |  |  | |  | |  | |
| 40–50 years | 0.15 | 0.34 | | 0.45 | | 1.08 | |
| 50–60 years | 1.32 | 1.2 | | 1.47 | | 2.78 | |
| 60–70 years | 4.96 | 4.18 | | 4.92 | | 7.96 | |
| ***Male*** |  |  | |  | |  | |
| Crude AF incidence |  |  | |  | |  | |
| 40–50 years | 1.35 | 1.12 | | 1.17 | | 2.18 | |
| 50–60 years | 2.81 | 2.86 | | 3.21 | | 6.06 | |
| 60–70 years | 11.13 | | 7.79 | | 9.32 | | 14.47 |

^a^sex-adjusted AF incidence (/1,000 PYRs)

**Supplementary Table S7. Baseline Characteristics in Subject Aged < 40 and >= 70 years.**

|  | **Korean NHIS-HealS** | **U.K. Biobank** | *P* value |
| --- | --- | --- | --- |
|  | **(n = 49,998)** | **(n = 9,607)** |  |
| Age, years | 73.8 ± 3.6 | 70.0 ± 0.6 | <0.001 |
| Male | 23236 (46.5) | 4730 (49.2) | <0.001 |
| BMI, kg/m^2^ | 23.5 ± 3.2 | 27.5 ± 4.3 | <0.001 |
| BMI categories |  |  | <0.001 |
| Underweight (<18.5 kg/m^2^) | 2741 (5.5) | 51 (0.5) |  |
| Normal (18.5 to < 25.0 kg/m^2^) | 32043 (64.1) | 2726 (28.4) |  |
| Overweight (25.0 to < 30.0 kg/m^2^) | 13976 (28.0) | 4556 (47.4) |  |
| Obese (≥30.0 kg/m^2^) | 1238 ( 2.5) | 2274 (23.7) |  |
| WC, cm | 82.6 ± 8.2 | 92.0 ± 12.5 | <0.001 |
| Abdominal obesity | 17798 (42.2) | 7131 (74.2) | <0.001 |
| SBP, mmHg | 132.8 ± 18.0 | 147.7 ± 19.2 | <0.001 |
| DBP, mmHg | 79.3 ± 10.9 | 81.6 ± 10.1 | <0.001 |
| Smoking | 5404 (11.5) | 2914 (31.9) | <0.001 |
| Alcohol | 8143 (16.3) | 121 (7.2) | <0.001 |
| Heart failure | 4862 (9.7) | 356 (3.7) | <0.001 |
| Hypertension | 30529 (61.1) | 2347 (24.4) | <0.001 |
| Diabetes mellitus | 7453 (14.9) | 814 ( 8.5) | <0.001 |
| Ischemic stroke or TIA | 6854 (13.7) | 385 ( 4.0) | <0.001 |
| Previous MI | 1397 ( 2.8) | 66 ( 0.7) | <0.001 |
| Hyperthyroidism | 1377 ( 2.8) | 107 ( 1.1) | <0.001 |
| Hypothyroidism | 1333 ( 2.7) | 682 ( 7.1) | <0.001 |
| Osteoporosis | 16016 (32.0) | 490 ( 5.1) | <0.001 |
| Dyslipidemia | 16869 (33.7) | 2556 (26.6) | <0.001 |
| ESRD or CKD | 719 (1.4) | 260 (2.7) | <0.001 |
| COPD | 4889 (9.8) | 112 (1.2) | <0.001 |
| History of malignant neoplasm | 6475 (13.0) | 1556 (16.2) | <0.001 |
| Glucose, mmol/L | 102.1 ± 30.8 | 95.4 ± 22.7 | <0.001 |
| Creatinine, mg/dL | 1.0 ± 0.8 | 0.9 ± 0.2 | <0.001 |
| Cholesterol, mg/dL | 197.6 ± 38.8 | 214.2 ± 47.6 | <0.001 |
| Triglycerides, mg/dL | 142.2 ± 84.1 | 154.8 ± 79.1 | <0.001 |
| LDL, mg/dL | 117.0 ± 36.4 | 132.6 ± 35.7 | <0.001 |
| HDL, mg/dL | 53.6 ± 29.5 | 56.0 ± 15.1 | <0.001 |

Abbreviations: NHIS-HealS, National Health Insurance Service’s Health Screening.

**Supplementary Table S8. Crude and Adjusted Incidence Rates of AF by Abdominal Obesity**

|  | | | **Korean NHIS-HealS** | | | **U.K. Biobank** | |  |
| --- | --- | --- | --- | --- | --- | --- | --- | --- |
|  | **Abdominal obesity (-)** | | | **Abdominal obesity (+)** | **Abdominal obesity (-)** | | **Abdominal obesity (+)** | |
|  | **(n = 261,536)** | | | **(n = 121,006)** | **(n = 158,049)** | | **(n = 319,774)** | |
| ***Overall*** | |  | |  |  | |  | |
| Numbers of events/PYRs | | 4,916/1,916,522 | | 3,205/876,422 | 4,703/1,832,541 | | 18,812/3,636,400 | |
| AF incidence (/1,000 PYRs) | |  | |  |  | |  | |
| Age- and sex-adjusted | | 2.64 | | 3.72 | 3.13 | | 4.92 | |
| Crude AF incidence (/1,000 PYRs) ^a^ | |  | |  |  | |  | |
| 40–50 years | | 1.42 | | 2.08 | 0.57 | | 1.17 | |
| 50–60 years | | 2.34 | | 3.05 | 1.62 | | 3.13 | |
| 60–70 years | | 5.22 | | 5.65 | 5.14 | | 8.52 | |
| ***Female*** | |  | |  |  | |  | |
| Numbers of events/ PYRs | | 1,383/714,540 | | 1,666/547,759 | 2,233/1,190,341 | | 6,758/1,855,415 | |
| AF incidence (/1,000 PYRs) | |  | |  |  | |  | |
| Age-adjusted | | 2.17 | | 2.74 | 2.13 | | 3.47 | |
| Crude AF incidence (/1,000 PYRs) | |  | |  |  | |  | |
| 40–50 years | | 1.10 | | 1.54 | 0.32 | | 0.72 | |
| 50–60 years | | 1.81 | | 2.35 | 1.14 | | 2.03 | |
| 60–70 years | | 3.88 | | 4.64 | 3.91 | | 6.12 | |
| ***Male*** | |  | |  |  | |  | |
| Numbers of events/ PYRs | | 3,533/1,201,982 | | 1,539/328,662 | 2,470/642,200 | | 12,054/1,780,986 | |
| AF incidence (/1,000 PYRs) | |  | |  |  | |  | |
| Age-adjusted | | 3.03 | | 4.53 | 4.35 | | 6.70 | |
| Crude AF incidence (/1,000 PYRs) | |  | |  |  | |  | |
| 40–50 years | | 1.61 | | 2.63 | 1.00 | | 1.59 | |
| 50–60 years | | 2.68 | | 4.25 | 2.60 | | 4.32 | |
| 60–70 years | | 5.97 | | 8.12 | 7.33 | | 11.05 | |

Abbreviations: NHIS-HealS, National Health Insurance Service’s Health Screening; PYRs, person-years.

^a^sex-adjusted AF incidence

**Supplementary Table S9. AF Risk of WC according to the Age-, Sex-, and Clinical Variable–adjusted Model**

|  | **Korean NHIS-HealS** | | **U.K. Biobank** | | ***P* for interaction** |
| --- | --- | --- | --- | --- | --- |
|  | **sHR (95% CI)** | ***P* value** | **sHR (95% CI)** | ***P* value** |  |
| ***With WC as a 1-SD increase*** | 1-SD (7.8-cm) |  | 1-SD (13.4-cm) |  |  |
| Age, sex adjusted | 1.22 (1.20–1.25) | <0.001 | 1.46 (1.44–1.48) | <0.001 | <0.001 |
| Adjusted for clinical variables ^a^ | 1.14 (1.11–1.17) | <0.001 | 1.35 (1.30–1.39) | <0.001 | <0.001 |
| ***With WC as a categorical variable*** |  |  |  |  |  |
| Age, sex adjusted |  |  |  |  |  |
| Abdominal obesity (-) | 1 (reference) |  | 1 (reference) |  |  |
| Abdominal obesity (+) | 1.36 (1.30–1.43) | <0.001 | 1.56 (1.51–1.61) | <0.001 | <0.001 |
| Adjusted for clinical variables ^a^ |  |  |  |  |  |
| Abdominal obesity (-) | 1 (reference) |  | 1 (reference) |  |  |
| Abdominal obesity (+) | 1.21 (1.14–1.27) | <0.001 | 1.32 (1.24–1.42) | <0.001 | <0.001 |

^a^ Adjusted for age, sex, and clinical variables including smoking, alcohol, heart failure, hypertension, diabetes mellitus, ischemic stroke or transient ischemic attack, previous myocardial infarction, hyperthyroidism, hypothyroidism, osteoporosis, dyslipidemia, end stage renal disease or chronic kidney disease, chronic obstructive pulmonary disorder, and history malignant neoplasm.

Abbreviations: NHIS-HealS, National Health Insurance Service’s Health Screening; sHR, subdistribution hazard ratio.

**Supplementary Table S10. Adjusted Incidence of AF by BMI in Subject Aged < 40 and ≥ 70 years.**

|  | **Korean NHIS-HealS cohort** | | | |
| --- | --- | --- | --- | --- |
|  | **Underweight**  **(n=2,741)** | **Normal-weight**  **(n=32,043)** | **Overweight**  **(n=13,976)** | **Obese**  **(n=1,238)** |
| ***Overall*** |  |  |  |  |
| Numbers of events/PYRs | 163/15,906 | 2,104/206,394 | 1,024/91,868 | 101/7,993 |
| AF incidence (/1,000 PYRs) ^a^ | 9.98 | 10.15 | 11.66 | 14.71 |
| ***Female*** |  |  |  |  |
| Numbers of events/PYRs | 65/7,521 | 909/104,283 | 561/57,737 | 76/6,456 |
| AF incidence (/1,000 PYRs) ^b^ | 8.61 | 8.75 | 9.88 | 11.70 |
| ***Male*** |  |  |  |  |
| Numbers of events/PYRs | 98/8,385 | 1,195/102,111 | 463/34,132 | 25/1,536 |
| AF incidence (/1,000 PYRs) ^b^ | 11.57 | 11.77 | 13.72 | 18.17 |
|  | **U.K. Biobank** | | | |
|  | **Underweight**  **(n=51)** | **Normal-weight**  **(n=2,726)** | **Overweight**  **(n=4,556)** | **Obese**  **(n=2,274)** |
| ***Overall*** |  |  |  |  |
| Numbers of events/PYRs | 6/489 | 303/29,282 | 623/48,284 | 428/23,291 |
| AF incidence (/1,000 PYRs) ^a^ | 15.07 | 10.70 | 12.66 | 18.84 |
| ***Female*** |  |  |  |  |
| Numbers of events/PYRs | 4/389 | 148/17,204 | 213/22,816 | 175/12,861 |
| AF incidence (/1,000 PYRs) ^b^ | 10.29 | 8.60 | 9.34 | 13.60 |
| ***Male*** |  |  |  |  |
| Numbers of events/PYRs | 2/100 | 155/12,078 | 410/25468 | 253/10,431 |
| AF incidence (/1,000 PYRs) ^b^ | 20.00 | 12.86 | 16.09 | 24.24 |

Abbreviations: NHIS-HealS; National Health Insurance Service-Health Screening; PYRs, person-years.

^a^ sex- and age-adjusted AF incidence

^b^ age-adjusted AF incidence

**Supplementary Table S11. Adjusted AF Risk according to BMI in Subject Aged < 40 and ≥ 70 years.**

|  | **Korean NHIS-HealS** | | **U.K. Biobank** | | ***P-*for interaction** |
| --- | --- | --- | --- | --- | --- |
|  | **sHR (95% CI)** | ***P* value** | **sHR (95% CI)** | ***P* value** |  |
| ***With BMI as a 1-SD increase*** | 1-SD (3.2 kg/m^2^) |  | 1-SD (4.3 kg/m^2^) |  |  |
| Age, sex-adjusted | 1.11 (1.08–1.15) | <0.001 | 1.25 (1.19–1.32) | <0.001 | 0.923 |
| Adjusted for clinical variables ^a^ | 1.06 (1.02–1.10) | 0.002 | 1.20 (1.13–1.26) | <0.001 | 0.029 |
| ***With BMI as a categorical variable*** |  |  |  |  |  |
| Age, sex adjusted |  |  |  |  |  |
| Underweight (<18.5 kg/m^2^) | 0.92 (0.82–1.02) | 0.105 | 1.20 (0.54–2.68) | 0.658 | 0.030 |
| Normal (18.5 kg/m^2^ to <25 kg/m^2^) | 1 (reference) |  | 1 (reference) |  |  |
| Overweight (25 kg/m^2^ to <30 kg/m^2^) | 1.26 (1.21–1.31) | <0.001 | 1.18 (1.03–1.35) | 0.018 | <0.001 |
| Obese (≥30 kg/m^2^) | 1.72 (1.56–1.88) | <0.001 | 1.73 (1.46–1.81) | <0.001 | <0.001 |
| Adjusted for clinical variables ^a^ |  |  |  |  |  |
| Underweight (<18.5 kg/m^2^) | 0.87 (0.74–1.03) | 0.105 | 0.80 (0.45–1.45) | 0.633 | 0.625 |
| Normal (18.5 kg/m^2^ to <25 kg/m^2^) | 1 (reference) |  | 1 (reference) |  |  |
| Overweight (25 kg/m^2^ to <30 kg/m^2^) | 1.13 (1.04–1.22) | 0.002 | 1.12 (0.98–1.30) | 0.088 | 0.173 |
| Obese (≥30 kg/m^2^) | 1.32 (1.07–1.62) | 0.009 | 1.52 (1.30–1.77) | <0.001 | 0.032 |

Abbreviations: NHIS-HealS, National Health Insurance Service-Health Screening; sHR, Subdistribution hazard ratio.

^a^ Adjusted for age, sex, and clinical variables including smoking, alcohol use, heart failure, hypertension, diabetes mellitus, ischemic stroke or transient ischemic attack, previous myocardial infarction, hyperthyroidism, hypothyroidism, osteoporosis, dyslipidemia, end stage renal disease or chronic kidney disease, chronic obstructive pulmonary disorder, and history malignant neoplasm.

**Supplementary Figures Legend**

**Supplementary Figure S1. The cumulative incidence of AF in participant without and with abdominal obesity in the Korean NHIS-HealS (A) and U.K. Biobank (B) cohorts.** Patients with abdominal obesity had a greater cumulative incidence of new-onset AF than those without abdominal obesity in both cohorts


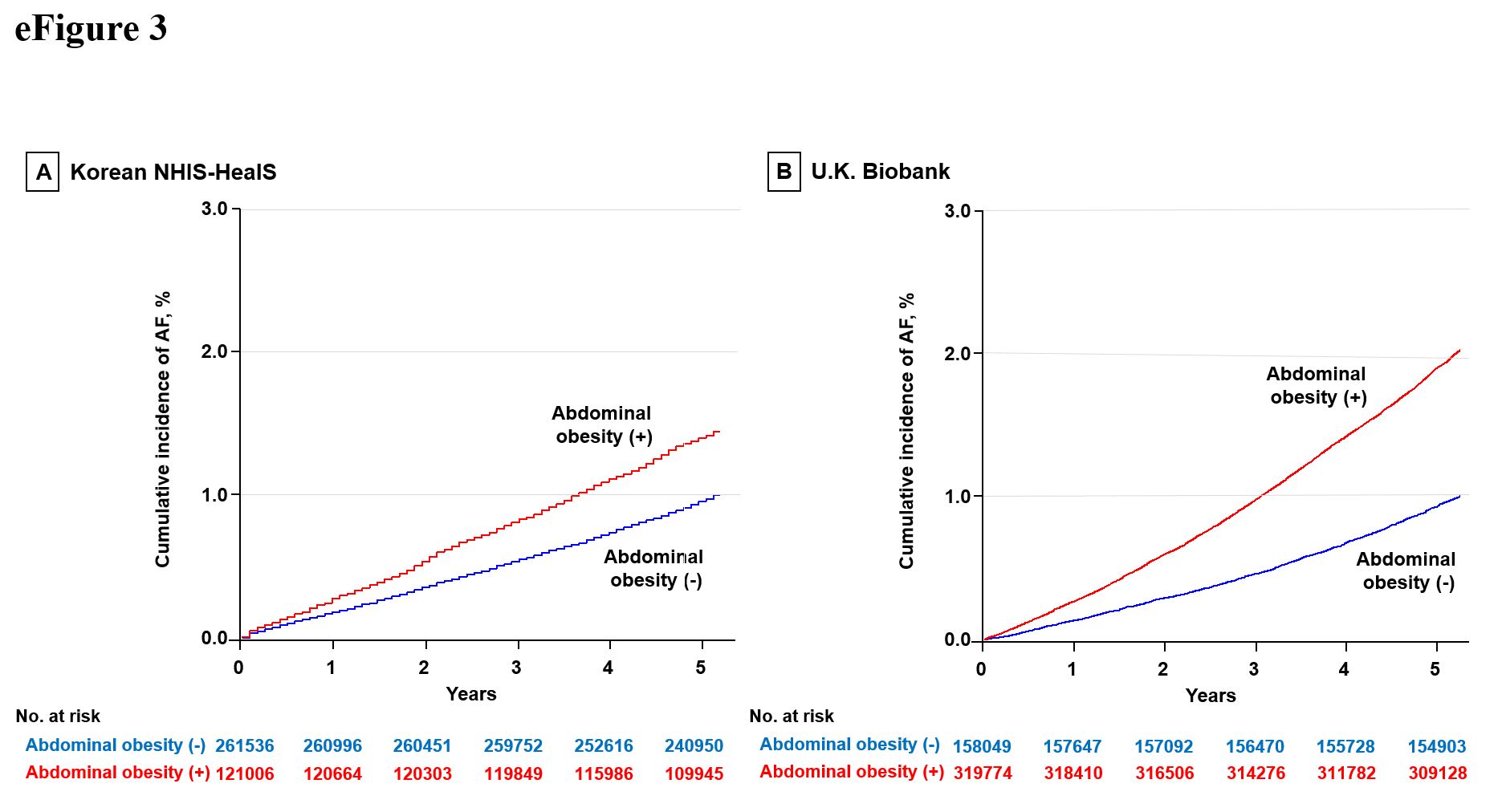

Supplement: Supplementary file 1 — Supplementary Information. [file 41598_2023_32229_MOESM1_ESM.docx]
